# Preoperative Magnetic Resonance Imaging Abnormalities Predict Symptomatic Adjacent Segment Degeneration After Anterior Cervical Discectomy and Fusion

**DOI:** 10.7759/cureus.17282

**Published:** 2021-08-18

**Authors:** Bornali Kundu, Ilyas Eli, Andrew Dailey, Lubdha M Shah, Marcus D Mazur

**Affiliations:** 1 Department of Neurosurgery, University of Utah, Salt Lake City, USA; 2 Department of Radiology, University of Utah, Salt Lake City, USA

**Keywords:** adjacent segment degeneration, adjacent segment disease, anterior, anterior cervical discectomy and fusion, cervical spine, magnetic resonance imaging, reoperation

## Abstract

Introduction

Anterior cervical discectomy and fusions (ACDFs) are generally limited to the levels causing neurological symptoms, but whether adjacent asymptomatic levels should be included if they demonstrate severe radiographic degeneration is a matter of controversy. We evaluated whether asymptomatic preoperative magnetic resonance imaging (MRI) abnormalities at adjacent levels were predictive of reoperation for symptomatic adjacent-segment degeneration (ASD) after the initial ACDF.

Methods

We reviewed patients treated with ACDF in 2000-2010 who had MRIs preoperatively and again ≥3 years after the index surgery to evaluate new neurological symptoms. Patients were stratified by ASD severity score, calculated based on MRI features. The associations between preoperative ASD severity score and reoperation for ASD were evaluated with logistic and Cox regressions after adjusting for covariates.

Results

Of 1038 patients who underwent ACDF, 96 (9%) had MRI evaluation ≥3 years postoperatively (mean follow-up 78 months). Of the 195 adjacent segments evaluated, 14 (7%) were included in subsequent fusion procedures. The 10-year surgery-free survival estimate was 82.7% (73.4-93.2%). After adjusting for covariates, ASD severity scores were predictive of reoperation only for patients with the highest score (hazard ratio [HR] 4.5 [1.0-19.8]) and those with foraminal stenosis (HR 4.2 [.4-12.7]). However, the prevalence of reoperation for ASD in these groups was only 16% and 15%, respectively.

Conclusion

The prevalence of reoperation for ASD was low for patients who presented with new symptoms ≥3 years after the index ACDF. Our findings do not support including asymptomatic levels in an anterior fusion construct, even if severe MRI abnormalities are present preoperatively.

## Introduction

Age-related degenerative changes of the cervical spine are present in many individuals despite the absence of symptoms. More than 60% of healthy, asymptomatic subjects ≥40 years of age have abnormal findings on magnetic resonance imaging (MRI) [1]. It is also widely believed that degeneration is accelerated by an anterior cervical discectomy and fusion (ACDF) of adjacent segments [2]. Although only a proportion of patients who develop adjacent-segment degeneration (ASD) will have symptoms at the adjacent segments, severe MRI abnormalities in the adjacent segments of a planned fusion construct are common [3]. Longitudinal studies have demonstrated an association between ASD and the development of new neurological symptoms [4], but the relationship between preoperative MRI abnormalities and the need for additional surgery at adjacent segments is not clear. Moreover, the question remains whether there are risk factors that may predict which patients are most likely to develop symptomatic ASD and require surgery.

Anterior fusion operations are generally limited to the levels that are thought to be the cause of radiculopathy or myelopathy [5]. Although asymptomatic adjacent segments may be included in an anterior construct if severe radiographic abnormalities are present, there is little evidence to predict the benefit of preemptively fusing a segment that is not contributing to the patient’s neurological symptoms. The ability to identify whether levels adjacent to the planned fusion are at high risk for symptomatic degeneration and then include them at the index operation could reduce risks associated with repeat surgery, diminish cost, and reduce time off from work, for example, for the patient. Our objective was to determine whether patients with preoperative MRI abnormalities at the level above or below the planned fused segment are at increased risk for undergoing reoperation for symptomatic ASD after ACDF.

## Materials and methods

Patient selection

A search of the operative database at our institution identified all patients who underwent ACDF operations performed by surgeons in the neurosurgery department from January 2000 to December 2010. To identify patients with symptomatic ASD, we included those who had a cervical spine MRI ≥3 years after the index operation for new or recurrent radiculopathy, myelopathy, and neck pain. Patients who had revision surgery for persistent pain or insufficient treatment <3 years after the index operation were excluded because we wanted to exclude those in whom MRIs were done to address perioperative complications unrelated to the development of ASD.

Grading ASD

Adjacent segments were defined as the next level above or below the fused segment. T2-weighted sequences in sagittal and axial views for each patient were evaluated for degeneration. Each adjacent segment was assessed using a minor modification of previously published MRI criteria (Table 1) [3,6]. Five separate components were graded: disk signal intensity (DSI), anterior compression of the spinal cord (AC), posterior disc protrusion (PDP), disc space narrowing (DSN), and foraminal stenosis (FS). Points were assigned according to the extent of degeneration for each component. FS was considered present if there was ≥50% narrowing on the axial section, a criterion that has demonstrated high inter-rater reliability [7]. A total ASD severity score was calculated for each level, ranging from 0 to 11. A board-certified fellowship-trained neuroradiologist who was blinded to whether the patient underwent reoperation graded the index surgery preoperative MRIs.

**Table 1:** Assessment criteria for adjacent-segment degeneration on magnetic resonance imaging of the cervical spine [3,6]

Finding	Severity score	Description
Decrease in disk signal intensity of intervertebral disc	0	Bright as or slightly less bright than cerebrospinal fluid
	1	Markedly darker than cerebrospinal fluid
	2	No signal
Anterior compression of dura and spinal cord	0	No compression
	1	Compression on dural sac only
	2	Compression on less than one-third of spinal cord
	3	Compression on more than one-third of spinal cord
	4	Compression on more than two-thirds of spinal cord
Posterior disc protrusion	0	No protrusion
	1	Protrusion beyond vertebral body without cord compression
	2	Protrusion beyond vertebral body with cord compression
Disc space narrowing	0	100–76% of height of upper healthy disc
	1	75–50% of height of upper healthy disc
	2	<50% of height of upper healthy disc
Foraminal stenosis	0	<50% narrowing of neural foramen
	1	≥50% narrowing of neural foramen
Sum total possible score	11	

Statistical analysis

Statistical analysis was performed using R software (version 3.0.2, R Foundation for Statistical Computing, Vienna, Austria, http://www.R-project.org). Inter-rater reliability of the components of the ASD score was calculated using one-way single-score intra-class correlation coefficients on a subset of random 80 samples.

We examined whether the main predictor variable of interest, the ASD severity score on preoperative MRI for the index case, was associated with the need for reoperation. We also evaluated potential confounding risk factors, including age, sex, number of levels fused, location of levels fused (superior or inferior to the fusion construct), and the length of time between index surgery and postoperative, follow-up MRI (which must be ≥3 years). Chi-square and Student’s t-tests were used for univariate analysis to identify potential predictors of adjacent segment fusion procedures ≥3 years after the index ACDF. Variables of interest and those with *p* ≤ 0.10 were included in a multivariate logistic regression analysis to test for the association between preoperative ASD score and reoperation while controlling for possible confounders. Cox and logistic regression analyses were used to adjust for the time-dependent variables (age and length of follow-up) and time-independent variables, respectively.

For Kaplan-Meier survival analysis, patients were grouped according to whether reoperation was performed, using the time to reoperation for causal outcome and time to follow-up MRI otherwise. Subjects were further stratified by ASD score into three groups: 0-1, 2-4, and ≥5. Patients who had a follow-up MRI at ≥3 years but no operation were censored data. A sub-analysis was done using FS and AC as predictors of ASD severity score because it is unlikely that patients without neural compression would be offered surgery at our institution, and FS is a strong predictor of the surgical level and adjacent level for ACDF operations in the literature [8].

## Results

Patient characteristics and surgical details

**Figure 1:**
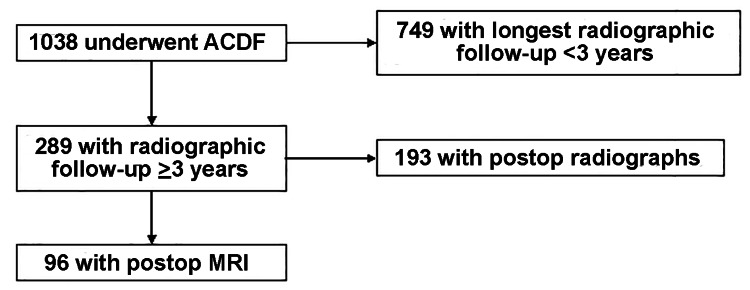
Flow chart of patient inclusion criteria

Of 1038 patients who underwent an ACDF (mean age 50 years, range 25-76 years), 96 (9%) had a cervical spine MRI ≥3 years after the index operation to evaluate new or recurrent radiculopathy, myelopathy, and neck pain (Figure 1). The mean follow-up was 78 months (range 36-161). There were 47 1-level, 37 2-level, 10 3-level, and 2 4-level index ACDF operations done. Three patients had skip-level ACDFs, with an unfused segment between the fused levels, resulting in three adjacent segments per index ACDF for evaluation. In total, there were 195 segments adjacent to the fusion available for assessment.

The inter-observer reliability of the ASD scoring system was moderate to substantial (intraclass correlation 0.70; 95% confidence interval [CI] 0.61-0.78). The mean (± standard deviation [SD]) ASD scores on pre- and ≥3-year postoperative MRI were 2.5 ± 2.0 (range 0-9) and 3.8 ± 2.3 (0-10), respectively. For each component of the ASD score, mean pre- and ≥3-year postoperative scores were DSI 0.8 ± 0.6 and 1.0 ± 0.5, AC 0.6 ± 0.7 and 0.9 ± 0.8, PDP 0.7 ± 0.6 and 0.9 ± 0.7, DSN 0.2 ± 0.5 and 0.6 ± 0.8, and FS 0.2 ± 0.4 and 0.4 ± 0.5, respectively, showing disease progression over time.

Reoperation rate for ASD

Fourteen segments (from 10 patients) of the 195 segments adjacent to the index ACDF (7%) underwent reoperation (Table 2). Of these, 86% (12/14) of the adjacent-segment surgeries occurred >5 years after the index ACDF surgery. Seven patients had reoperation of a 1-level ACDF at one adjacent segment. Multilevel posterior fusion reoperations were done in the other seven segments (two patients at two adjacent segments and one patient at three adjacent segments). No patients underwent posterior foraminotomy procedures.

**Table 2:** Surgical details of adjacent-segment operations ACDF, anterior cervical discectomy and fusion; ASD, adjacent-segment degeneration; FS, foraminal stenosis; PSF, posterior instrumented spinal fusion.

Patient #	Level #	Levels included in index ACDF	Preoperative ASD severity score	Preoperative FS present?	Length of follow-up (months)	Postoperative FS status	Levels included in adjacent-level surgery
1	1	C5-6, C6-7	1	Yes	38	Stable	C7-T1 ACDF
2	2	C4-5, C5-6	1	No	69	Worse	C6-7 ACDF
3	3	C6-7	5	Yes	74	Worse	C5-6 ACDF
4	4	C5-6	6	Yes	41	Stable	C6-7 ACDF
5	5	C5-6	6	No	77	Worse	C4-7 PSF
	6	C5-6	4	No	77	Worse	C4-7 PSF
6	7	C4-5, C5-6, C6-7	1	No	150	Worse	C3-4 ACDF
7	8	C3-4	4	No	64	Worse	C2-5 PSF
	9	C3-4	9	Yes	64	Stable	C2-5 PSF
8	10	C3-7	5	No	79	None	C2-3 ACDF
9	11	C4-5, C6-7	3	No	155	None	C3-T1 PSF
	12	C4-5, C6-7	4	No	155	None	C3-T1 PSF
	13	C4-5, C6-7	1	No	155	None	C3-T1 PSF
10	14	C5-6	3	Yes	107	Worse	C4-5 ACDF

Table 3 depicts the frequency of reoperation by preoperative ASD severity. The prevalence of adjacent-segment surgery was low even among subjects with the highest severity score: 16% for ASD ≥5, 7% for ASD 2-4, and 4% for ASD 0-1 (Table 3). 

**Table 3:** Frequency of reoperation at adjacent segment by preoperative ASD score ASD, adjacent-segment degeneration.

ASD score (total points)	0	1	2	3	4	5	6	7	8	9
Number of levels with reoperation	0	3	1	1	4	2	2	0	0	1
Number of levels with no reoperation	34	40	20	44	17	14	7	2	2	1

**Figure 2:**
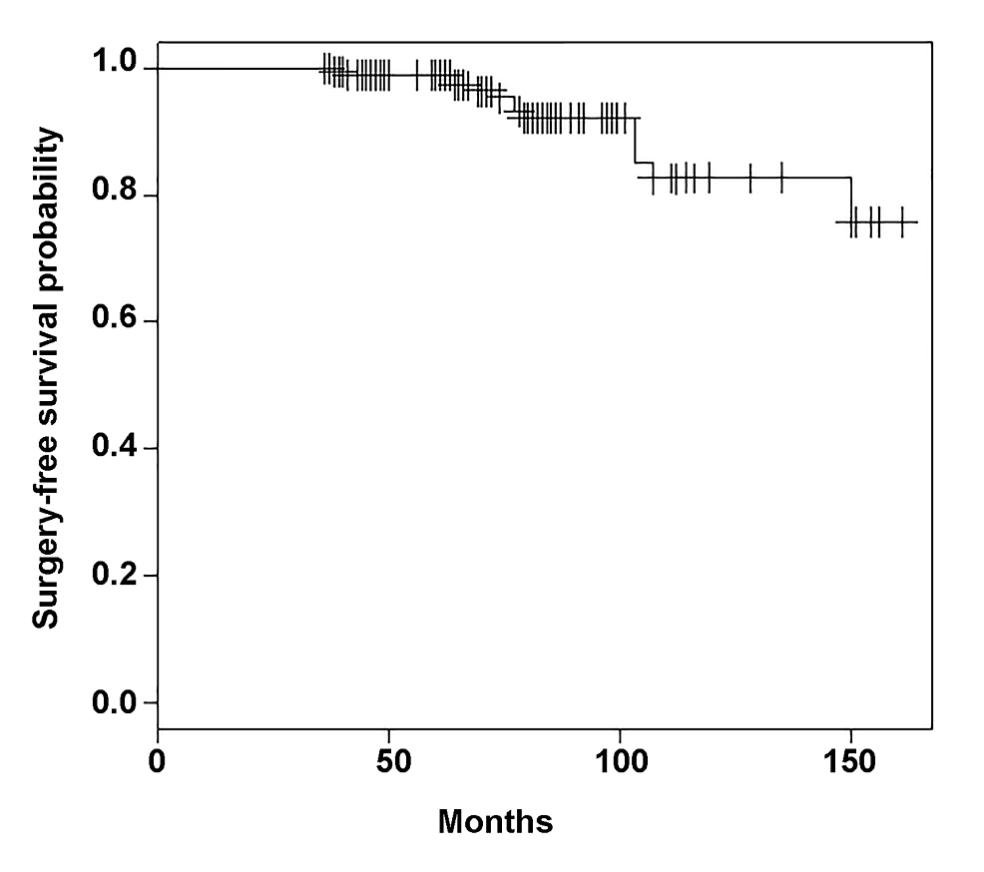
Kaplan-Meier estimates of adjacent-segment surgery-free survival after anterior cervical discectomy and fusion Kaplan-Meier estimates of surgery-free survival were 98.9% (95% CI 97.3-100%) at 5 years, 92.1% (87.2-97.4%) at 8 years, and 82.7% at 10 years (73.4-93.2%). CI, confidence interval.

Kaplan-Meier estimates of adjacent-segment surgery-free survival were 98.9% (95% CI 97.3-100%) at 60 months, 92.1% (87.2-97.4%) at 96 months, and 82.7% at 120 months (73.4-93.2%) (Figure 2).

Predictors of adjacent-segment surgery

Segments that required reoperation had higher ASD severity scores on preoperative MRI than those that did not require reoperation (3.9 ± 2.3 vs. 2.4 ± 1.9, respectively; *p *= 0.03). Within a multivariate logistic regression analysis, preoperative ASD score (odds ratio [OR] 1.40 [95% CI 1.06-1.82]), *p *= 0.02] was independently predictive of adjacent-segment surgery after accounting for time between MRIs (*p *= 0.36), patient age at index operation (*p *= 0.55), number of levels fused (*p* = 0.95), and location of adjacent segment (*p *= 0.70) (Table 4).

**Table 4:** Logistic regression model of preoperative MRI abnormalities and potential cofounding risk factors on reoperation for adjacent-segment surgery after ACDF MRI, magnetic resonance imaging; ACDF, anterior cervical discectomy and fusion; CI, confidence interval; SD, standard deviation; OR, odds ratio.

Variable	Reoperation	No reoperation	Univariate *p*-value*	Multivariate	
				*p*-value*	OR (95% CI)
Mean age in yrs (±SD)	54.4 ± 12.1	50.0 ± 11.4	0.18	0.55	1.02 (0.96-1.07)
Follow-up time in months (±SD)	82.1 ± 29.3	77.1 ± 31.2	0.55	0.36	1.01 (0.99-1.03)
Mean no. levels fused (±SD)	1.7 ± 0.9	1.7 ± 0.7	0.82	0.95	1.02 (0.46-2.07)
Superior adjacent segment (%)	7 (50)	92 (51)	1.00	0.70	0.80 (0.25-2.54)
Mean preoperative ASD score (±SD)	3.9 ± 2.3	2.4 ± 1.9	0.03	0.02	1.40 (1.06-1.82)

We used Cox proportional hazards analysis to determine whether preoperative MRI abnormalities remained associated with reoperation after accounting for differences in length of follow-up and age. Subjects were stratified by ASD score into three groups: 0-1, 2-4, and ≥5. ASD score remained a predictor of reoperation after accounting for age and length of follow-up, but only for the group with the highest ASD scores. Using ASD 0 or 1 as the reference group, hazard ratios were 1.9 (95% CI 0.50-8.0, *p *= 0.27) for ASD 2-4, 4.5 (1.0-19.8, *p *= 0.05) for ASD ≥5, and 1.04 (0.98-1.09, *p *= 0.21) for age (log-rank *p *= 0.04).

**Figure 3:**
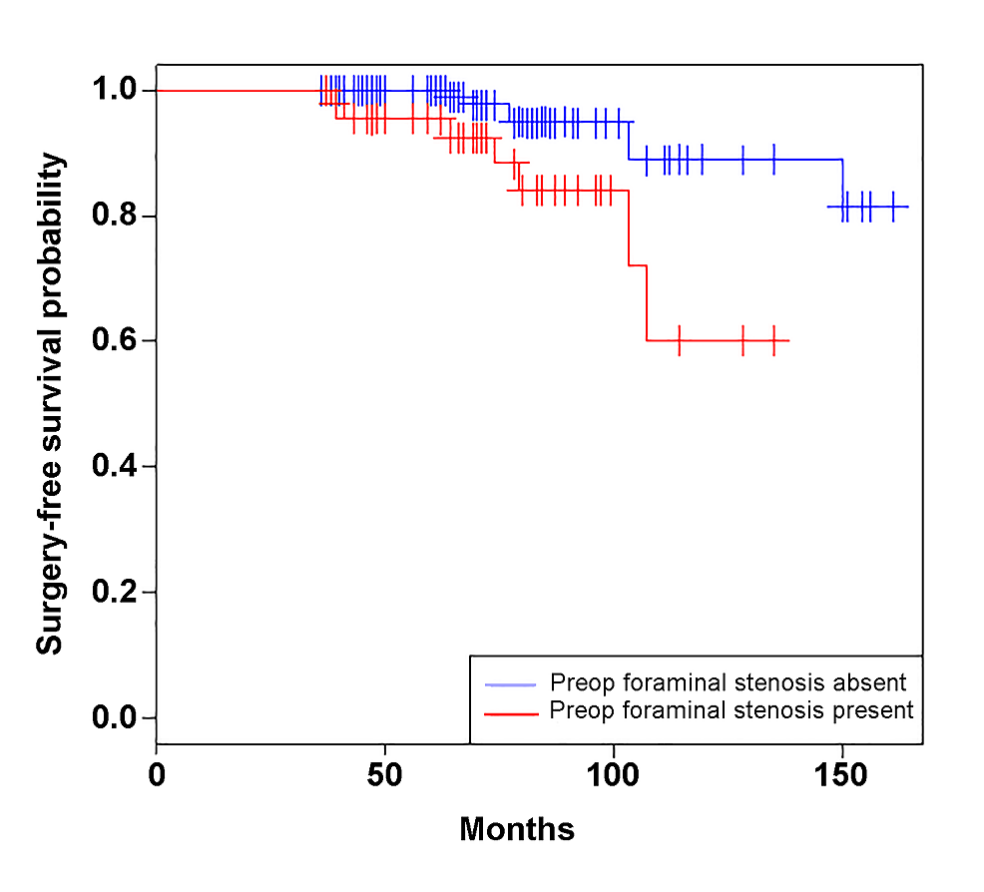
Kaplan-Meier estimates of adjacent-segment surgery-free survival after anterior cervical discectomy and fusion in patients with and without foraminal stenosis (FS) on preoperative magnetic resonance imaging Patients with preoperative FS had lower surgery-free survival than patients without preoperative FS (log-rank *p *< 0.01). After adjusting for age, the presence of preoperative FS remained a risk factor for adjacent-segment surgery on Cox proportional hazard analysis (HR 4.2 [95% CI 1.4-12.7], *p *= 0.01). HR, hazard ratio; CI, confidence interval.

In a subanalysis of patients with preoperative neural compression (FS or AC) on adjacent segments, preoperative FS was associated with reoperation on age-adjusted Cox regression analysis (hazard ratio [HR] 4.2 [1.4-12.7], Figure 3), but AC did not demonstrate this trend (2.6 [0.78-1.6]). Preoperative FS was present in 42% of segments (40/195 adjacent segments), but adjacent-segment surgery was performed only in 15% of these cases (7/40).

## Discussion

A current matter of debate is whether to include asymptomatic adjacent segments that look worrisome from an imaging standpoint into a fusion construct in an effort to prevent ASD-associated reoperation years into the future [2,4,9-12]. In a frequently cited paper, Hilibrand et al. [2] defined symptomatic ASD as new clinical symptoms that persisted for two consecutive follow-up visits, which allowed for the inclusion of patients managed both operatively and nonoperatively after the index operation. Interestingly, neither simple demographic factors such as age and body mass index, nor operation parameters such as graft type used in the index operation, symptoms at presentation, and the number of levels fused, predict the rate of reoperation for ASD [13]. Thus, current guidelines lack data to support “prophylactic” operations of adjacent segments [5], although current practitioners may consider including asymptomatic levels into an anterior fusion construct if MRI-based foraminal stenosis is significant.

In this study, we addressed whether quantifiable MRI-based parameters are predictive of reoperation at an adjacent segment after an index ACDF operation. We found that symptomatic ASD occurred in only 9% of patients who had an MRI to evaluate new neurological symptoms ≥3 years after the index ACDF. Fourteen of 196 (7%) adjacent levels were fused in 10 of 96 (10%) patients. We report a similar prevalence of reoperation for ASD in the available literature: 10-year surgery-free survival was 82.7% (95% CI 73.4-93.2%) in our study compared with 74.4% (68.4-80.4%) in the study by Hilibrand et al. [2]. Thus, 90% of patients presenting with symptoms that warranted MRI evaluation did not require reoperation. Our rate of symptomatic ASD is comparable with the 3.2% prevalence that was calculated using a pooled analysis of other studies with long-term (>5-year) follow-up [4]. Our rate of reoperation of 1% (10/1038) is also comparable with a previously published study that demonstrated a 1.4% (4/283) reoperation rate [9].

Considering these data, we caution against including asymptomatic levels in an anterior fusion construct, even if severe abnormalities are present on preoperative MRI. Although this population (those with new symptoms returning for an MRI ≥3 years after the index ACDF) may require reoperation (10%, 10/96 patients), after stratifying by the severity of preoperative MRI abnormalities, preoperative findings were only associated with reoperation in patients with the most severe abnormalities (ASD score ≥5). In this group, the prevalence of reoperation was only 16% (5/31 total levels assessed). In a subanalysis, this association was present for those with preoperative FS, of whom only 15% required reoperation. Thus, even patients with severe imaging abnormalities at the time of the index operation are likely to be managed nonoperatively at long-term follow-up. Two patients within our database had follow-up MRIs done <3 years after the index operation. Including them in the models did not significantly change the results.

Our study findings are relevant given the recent popularity of cervical disc arthroplasty, which is predicated upon the assertion that restoring physiologic kinematics of the cervical spine would decrease symptomatic ASD. Recent long-term data suggest superiority of cervical disc arthroplasty over ACDF for patient-reported outcomes measures, patient satisfaction, and additional surgery for ASD [14]. Over a 7-year period, 13 of 130 patients in the ACDF group (10%) underwent surgery for symptomatic ASD >2 years after the index procedure compared with 0 of 163 patients in the arthroplasty group. However, Kaplan-Meier curves comparing time to subsequent secondary surgical interventions (i.e., revision, removal, reoperation, or supplemental fixation) was not significantly different between the two groups (log-rank *p *= 0.123). Our low prevalence for adjacent-segment surgery (10%) suggests that ACDF remains a durable procedure and that revision surgery is usually not needed, even when patients develop subsequent neurological symptoms that warrant a follow-up MRI.

Interestingly, other MRI-based features such as measures of sagittal balance [9] and stable spondylolisthesis [15] have also not predicted ASD and the rate of reoperation. Considering the increased risk of complications associated with multilevel fusion that occur within 30 days of the operation [16], we advise against including asymptomatic levels with MRI-based abnormalities like measures of foraminal and canal stenosis and deformity. Unfortunately, it is not known whether ASD is related to the natural history of spondylolysis, by which patients with the existing disease may be at increased risk of symptomatic and progressive disease at an adjacent segment or elsewhere [10,11].

There are limitations to this retrospective cohort study. Selection bias is present because treatment was performed according to the surgeon’s discretion. Furthermore, the number of patients who may have undergone reoperation at another institution is not known. Patient-reported clinical outcomes data were not available for most of the patients during the study period, so we were not able to determine the relationship between obtaining a postoperative MRI and worsening disability and/or pain. Despite these limitations, our study provides data that may help surgeons counsel patients who return to follow-up with new symptoms years after an ACDF operation. Our results also inform larger prospective studies to evaluate the value of preoperative imaging parameters in predicting the need for adjacent-segment surgery.

## Conclusions

Reoperation for ASD was uncommon in this cohort of patients who had an MRI to evaluate neurological symptoms several years after undergoing ACDF. Our findings do not support including asymptomatic levels in an anterior fusion construct during the index operation in similar patients, even if severe radiographic abnormalities are present at those adjacent segments.
